# No Immunity From Having to Choose: An Exercise in Clinical Reasoning

**DOI:** 10.1007/s11606-025-09799-7

**Published:** 2025-09-12

**Authors:** Grace Yi, Paige Brown, Tyler Larsen, Kelley Chuang, Andrew S. Parsons, Inderpreet Saini, Satya Patel

**Affiliations:** 1 David Geffen School of Medicine at UCLA, 10833 Le Conte Ave, Los Angeles, CA 90095 USA; 2 West Los Angeles Veterans Affairs Hospital, Los Angeles, CA USA; 3 University of Virginia School of Medicine, Charlottesville, VA USA

**Keywords:** framing bias

**An 84-year-old male with hypertension, type 2 diabetes mellitus, stage III chronic kidney disease, hyperlipidemia, and benign prostatic hyperplasia presented to the hospital for an expedited workup of one week of painless dark brown urine and acute kidney injury (AKI), with recent outpatient renal ultrasound revealing a new 7.1-cm left renal mass.**

This elderly patient with several comorbidities predisposing him to kidney disease presents with acute on chronic kidney injury and a newly identified renal mass with painless dark urine. Dark urine is often equated with hematuria, but there are mimickers, including exogenous pigments from food or medications, or endogenous pigments like bilirubin, porphyrins, or myoglobin. A thorough workup includes careful review of urinalysis and urine microscopy to quantify the number of red blood cells and identify casts or dysmorphic red blood cells, which might suggest a glomerular cause.

While important to consider common causes of hematuria such as recent infection or nephrolithiasis, painless hematuria in this elderly patient also raises concern for malignancy along the urinary tract, particularly in the context of a newly diagnosed renal mass. If this renal mass represents malignancy, it is most likely a primary renal tumor such as renal cell carcinoma, as metastatic disease to the kidneys is rare.

As this patient’s acute on chronic kidney injury remains undifferentiated, timely characterization of the severity and acuity of his kidney injury is essential to address easily reversible pre-renal and post-renal causes. The absence of typical features of pre-renal and post-renal causes (e.g., signs of hypovolemia or volume overload, bladder fullness, and inability to micturate) as part of his key feature for hospitalization allows for prioritization of intrinsic causes of kidney injury. Utilizing an anatomical approach to intrarenal causes of acute on chronic kidney injury, including tubular, interstitial, glomerular, and vascular causes, can help ensure that all etiologies are considered. Acute tubular necrosis due to a yet unspecified insult is the most likely cause of intrinsic kidney injury, considering the base rate of disease prevalence. However, the presence of dark urine makes pigment nephropathy or glomerular disease worth considering. Maintaining simultaneous workups for his kidney injury and dark urine is important, as these problems could have a common cause or stem from two separate processes.

*The discussant uses the cognitive strategy of framing to develop an initial differential diagnosis. Framing is the clinician’s intentional selection of relevant details from the patient’s medical history or clinical presentation to formulate a problem representation. A clinician makes a choice to include or exclude information based on their knowledge base and personal experience with the illness or syndrome.*^1^ *This purposeful inclusion or exclusion of certain details can shift the problem representation drastically—either helping the clinician iteratively refine their problem representation until they reach the correct diagnosis or leading them toward an incorrect diagnosis.*^2^

*In this case, the initial problem representation begins with a broad descriptor (acute on chronic kidney injury in an elderly patient) and pointedly includes the recently identified renal mass and painless hematuria. Framing the clinical problem around painless hematuria in the presence of a renal mass allows the clinician to prioritize certain causes of intrinsic renal injury (e.g., pigment nephropathy and glomerular disease). The discussant also utilizes the principle of Hickam’s dictum—the idea that there may be as many simultaneous disease processes as there are patient concerns—to note that the patient’s kidney injury and dark urine may in fact be two unrelated processes.* ^3^

**He first noticed dark brown urine one week prior to presentation. There was no associated pain, though he had a chronically weak urinary stream secondary to BPH. He experienced six months of progressive fatigue and 40 pounds of unintentional weight loss. He did not experience any fevers, chills, night sweats, or abdominal pain. He had a 40-pack year smoking history (quit smoking 30 years prior). He did not report a history of alcohol or other substance use. His medications included atorvastatin, diltiazem, hydralazine, pantoprazole, and tamsulosin. He did not recently initiate any new medications, including non-steroidal anti-inflammatory drugs (NSAIDs), aspirin, or anticoagulants.**

This additional history clarifies the time course of his presentation. Subacute progressive fatigue and unintentional weight loss suggest a significant ongoing inflammatory process. His renal mass with painless hematuria is most concerning for malignancy. His remote smoking history is also a significant risk factor for urinary tract cancers such as bladder transitional cell carcinoma and renal cell carcinoma (RCC).

Even in the absence of overt fevers or other constitutional symptoms, other causes of chronic inflammation are also important considerations. These include indolent infections that can localize to the urinary tract such as tuberculosis or endemic fungi, autoimmune conditions such as anti-neutrophil cytoplasmic antibody (ANCA) vasculitis, or medication-related adverse effects such as hydralazine-induced lupus nephritis. The lack of overt infectious symptoms and abdominal pain render common causes of hematuria such as urinary tract infection or nephrolithiasis less likely. The patient also did not report taking NSAIDs or anticoagulants, which otherwise may have predisposed him to various forms of kidney injury and hematuria.

*The discussant uses the patient’s symptoms, older age, smoking history, and additional signs of unintentional weight loss and fatigue to prioritize malignancy within their differential diagnosis for chronic inflammatory processes. Additionally, the case is purposely framed as intrinsic kidney injury in the context of a chronic inflammatory process to accommodate the possibility of two simultaneous and unrelated diagnoses. The discussant considers indolent infection, autoimmune condition, or medication-related adverse effect, in addition to the existing concern for malignancy.*

**His vital signs in the emergency department were within normal limits. His hemoglobin was 12.7 g/dL (13.3 g/dL one month prior). His complete metabolic panel showed a sodium of 132 mEq/L and creatinine of 5.06 mg/dL, increased from 2.24 mg/dL one week prior (and overall increased from a baseline of 1.3 mg/dL). His urinalysis had 3 + blood and 2 + protein. Urine microscopy confirmed hyaline casts, greater than 1000 red blood cells/hpf, greater than 400 white blood cells/hpf, and no dysmorphic cells. Further urine studies revealed a microalbumin of 2109 mcg/mg and a protein to creatinine ratio of 4.4.**

These data confirm that the patient has significant acute on chronic intrinsic kidney injury and true hematuria accompanied by significant proteinuria and macroalbuminuria. While some degree of proteinuria can be expected in chronic kidney disease, the spot urine protein to creatinine ratio suggests nephrotic-range proteinuria. His clinical presentation of significant AKI and hematuria seems most consistent with a nephritic syndrome, but he does not have red blood cell casts or dysmorphic red blood cells as would be expected. A primary or secondary nephrotic syndrome seems even less likely without hypoalbuminemia or peripheral edema. Given the severity and acuity of his presentation, a kidney biopsy to examine for glomerulonephritis would be helpful. Further serologic evaluation of his possible glomerulonephritis would also be indicated, including complement levels, ANCA testing, and infectious serologies including Hepatitis B and C viruses.

*The findings from the patient’s urine studies represent a diagnostic pivot point. A diagnostic pivot point represents information revealed in a case that is often a distinguishing feature of a particular disease process. Selection of a diagnostic pivot point requires strategically emphasizing a few key results on which to focus, with attention to tempo of symptoms, while allowing other findings to remain in the background.* ^4^ *The discussant prioritizes the elevated serum creatinine and presence of hematuria over the degree of proteinuria and recognizes that dysmorphic red blood cells or red blood cell casts are not required to diagnose glomerulonephritis. This is a diagnostic pivot point that allows the clinician to focus their problem representation on nephritic syndrome. This helps the discussant focus on causes of glomerulonephritis and to draw on their management script to identify a high-yield test to confirm the diagnosis of glomerulonephritis and to consider a workup for underlying autoimmune disorders.*

**A magnetic resonance imaging (MRI) of the abdomen and pelvis with and without contrast revealed a 7.1-cm solid left lower pole renal mass posterior to the hilar vessels with features highly suggestive of clear cell renal cell carcinoma with no evidence of abdominal metastases (Fig.** **1** **). Computed tomography (CT) of the chest was obtained to evaluate for metastases and revealed upper lobe predominant ground glass opacities (GGOs), and interlobular septal thickening with bilateral small pleural effusions.**

**Figure 1:**
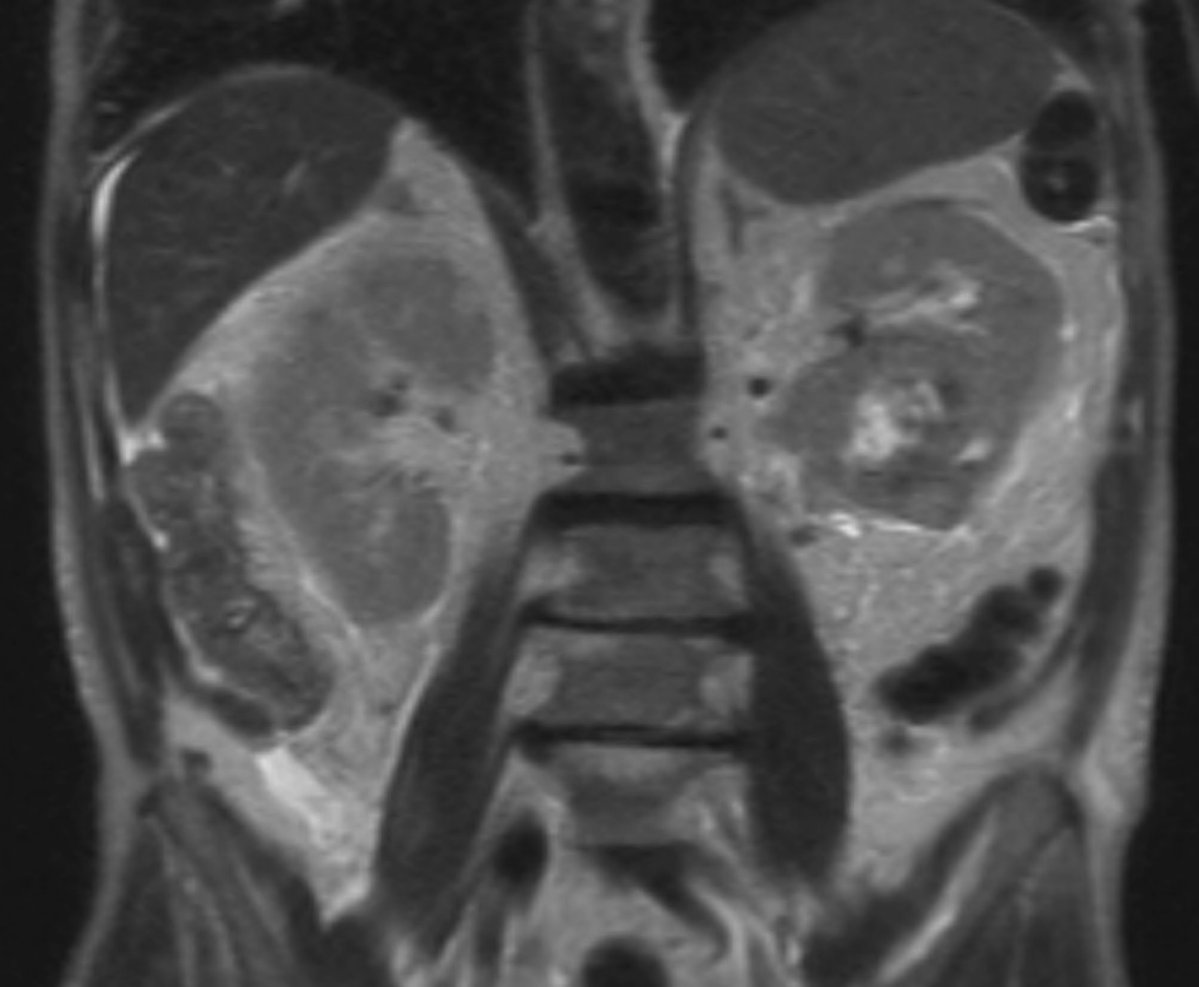
Magnetic resonance imaging (MRI) of the abdomen and pelvis with and without contrast revealing a 7.1-cm left lower pole renal mass.

The MRI confirms that the renal mass is likely RCC without obvious metastatic disease, which could certainly contribute to progressive weight loss and fatigue. However, RCC by itself does not fully explain his rapidly progressive glomerulonephritis and other symptoms, unless there is an associated paraneoplastic inflammatory process.

The presence of GGOs on his CT may represent pulmonary edema, most often caused by left-sided heart failure, but could represent infection or diffuse alveolar hemorrhage. In the context of subacute inflammation and acute glomerulonephritis, GGOs on CT alternatively raise concern for an inflammatory pulmonary-renal syndrome, including small vessel vasculitides such as anti-glomerular basement membrane disease or ANCA-associated vasculitis. These conditions may cause pulmonary edema and hemorrhage from capillary leakage. Further rheumatologic workup is warranted.

*The discussant focuses on the GGOs on CT of the chest, which is another pivot point in the case, as up until now there has been no mention of lung pathology. The discussant’s problem representation of “inflammatory pulmonary-renal syndrome” allows them to identify and navigate the diagnostic tension between malignancy and glomerulonephritis.* ^5^ *They continue to be aware of the opposing cognitive tools of Occam’s razor (one unifying diagnosis explains multiple complaints and clinical findings) and Hickam’s dictum, as they intentionally try to avoid premature closure on a unifying diagnosis to explain the patient’s findings.* ^3^

**Further laboratory workup revealed normal C3, borderline low C4 of 9 mg/dL, p-ANCA titer of > 1:1280, and elevated myeloperoxidase antibody of 114.5 CU. Rheumatology was consulted and recommended a right renal biopsy, which revealed focal necrotizing and crescentic glomerulonephritis with moderate interstitial scarring consistent with an early pauci-immune ANCA-associated glomerulonephritis (Fig.** **2** **). Hydralazine was discontinued as a possible inciting factor, and he was transitioned to an alternative anti-hypertensive agent. The patient received methylprednisolone for two days without improvement in renal function. This was stopped after he developed hyperglycemia requiring treatment with an insulin infusion. At this point, rheumatology considered rituximab for treatment of ANCA vasculitis, but first recommended hematology-oncology consultation to consider the potential impact of rituximab on the efficacy of immunotherapy (immune checkpoint inhibitors), which might be needed to treat his suspected RCC. Infectious diseases and pulmonology were consulted to comment on the GGOs seen on chest CT, which were ultimately attributed to pulmonary edema. Throughout this course, his renal function progressively worsened. He ultimately developed respiratory failure due to fluid overload that required initiation of intermittent hemodialysis (iHD). Repeat high-resolution CT of the chest showed an interval increase in the size of pleural effusions despite multiple iHD sessions.**

**Figure 2:**
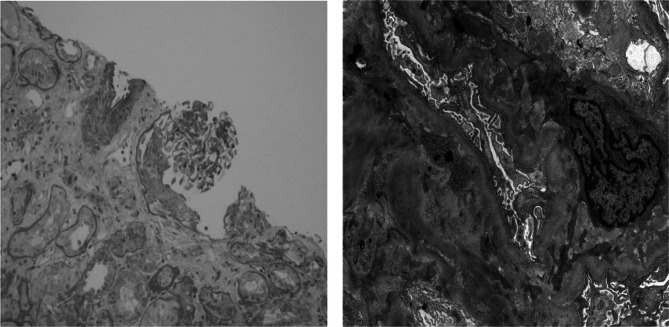
Small active crescent (L) and electron-dense deposits within the mesangium (R).

The high antibody titers and biopsy findings support the diagnosis of ANCA-associated vasculitis as the most likely cause of the patient’s rapidly progressive glomerulonephritis. A pattern of peripheral ANCA with myeloperoxidase antibodies is classically associated with microscopic polyangiitis; however, granulomatosis with polyangiitis, eosinophilic granulomatosis with polyangiitis, and drug-induced ANCA vasculitis are certainly possibilities to consider, especially given his exposure to hydralazine. The worsening pleural effusions and infiltrates despite iHD suggest there may be ongoing inflammation in the lungs at the level of the capillaries, which could be explained by a small vessel vasculitis such as ANCA vasculitis. In this setting, bronchoalveolar lavage may be helpful in evaluating for infection or diffuse alveolar hemorrhage, which is associated with a broad range of rheumatologic conditions.

The diagnosis of ANCA-associated vasculitis poses a management dilemma, as treatment of RCC may involve the use of immunotherapy while treatment of ANCA-associated vasculitis involves immunosuppression. If his RCC is confirmed to be early-stage, surgical resection may be curative. It is unclear if treatment for his RCC should be delayed in favor of treating his vasculitis and warrants further discussion with the patient.

*The clinician confirms the diagnosis of ANCA-associated vasculitis. They describe the conflicting management of RCC and ANCA-associated vasculitis and note the importance of a multidisciplinary patient-centered discussion to determine which condition should be prioritized in immediate treatment. This dilemma highlights the importance of management reasoning, the process of making testing and treatment decisions while incorporating a patient’s values and preferences and considering resource allocation.* ^6^ *The management script is a tool clinicians can use to organize the possible courses of action (e.g., labs, imaging, procedures, medications, consulting) they may take to address a patient’s diagnosis or syndrome.* ^7^ *In this case, management scripts may be activated as two illness-specific treatment menus: one for RCC and the other for ANCA-associated vasculitis.* ^8^ *As with any case, selecting from this menu of options is a prioritization task that requires consideration of the patient’s goals and values through shared decision-making, a risk–benefit analysis for each potential treatment, and grappling with the uncertainty inherent to this complex case.* ^9,10^ *Explicit consideration of the factors may improve decision-making. While diagnosis is inherently a categorization task with one or more right answers, in management the correct answer is almost always, “it depends.” The discussant accurately expresses the tension between prioritizing vasculitis over RCC, as treating one impacts treatment options for the other.*

**Pulmonology initially considered malignancy as the cause of his worsening pleural effusions. However, thoracentesis was deferred, as repeat CT of the chest demonstrated improvement in his bilateral pleural effusions and pulmonary edema with additional iHD. A presumptive diagnosis of RCC was made by the multidisciplinary team. Given the need to discuss the ramifications of treating RCC with immunotherapy in the context of active vasculitis, a family meeting was held. The patient elected to forego immunosuppression for treatment of his vasculitis in favor of nephrectomy for potential curative management of his RCC, with the understanding that he would likely require lifelong iHD. He successfully underwent partial left nephrectomy after hospital discharge, with negative margins and no nodal involvement on pathologic examination. He remained on iHD without appreciable renal recovery, though he continued to produce urine. Hematology-oncology subsequently recommended immunotherapy with pembrolizumab to decrease the risk of disease recurrence.**

**Unfortunately, after four doses of pembrolizumab, scans revealed metastatic recurrence to the patient’s lungs, mediastinal lymph nodes, and multiple intracranial areas. Axitinib was added to his regimen. Despite combined therapy, there were interval increases in the size of these metastatic lesions. Fifteen months after the initial hospitalization, the patient was readmitted to the hospital with toxic metabolic encephalopathy. At that time, the priority shifted towards comfort-oriented care, and the patient passed away during this hospital admission.**

## Discussion

This case illustrates the importance of iterative re-framing to gain diagnostic clarity and the challenges of navigating conflicting management pathways. This diagnosis relied heavily on laboratory and pathological data, but the tempo of illness and early confirmation of painless hematuria and proteinuria on urinalysis were pivotal in arriving at the diagnosis. In the end, timely identification of both the patient’s RCC and his ANCA-associated vasculitis was necessary to help the patient decide his optimal treatment path.

Patient goals, values, and preferences are important data points when navigating treatment decisions for parallel diagnoses with diverging management plans. Balancing the urgency of immunosuppression for ANCA-associated vasculitis against the patient’s choice for potentially curative surgery for RCC highlights how providing patient-centered care requires aligning management decisions with individual goals and values. In other words, understanding the patient’s multiple illnesses in the context of their overall condition was key to formulating a patient-centered management plan. In this case, the clinician is tasked with integrating separate management scripts into a unified management approach that considers potential cross-impacts, such as how immunosuppression could interfere with the efficacy of cancer treatment. The clinician needed to pause, conduct a risk–benefit analysis with a multidisciplinary team of consultants, and elicit the patient’s treatment preferences regarding renal replacement therapy, immunosuppression, and treatment of his malignancy. This patient’s journey was shaped by input from nephrology, rheumatology, oncology, pulmonology, and critical care teams. This case highlights the diversity in management pathways for which clinicians must engage in shared decision-making, particularly when options carry risks and benefits that are closely matched. Here, the patient’s values and preferences became central, as his choice to prioritize surgery reflected his understanding of the irreversible impacts of lifelong hemodialysis and the limitations of treating metastatic RCC with active vasculitis.

Effective management reasoning in cases with dual pathology involves integrating clinical judgment, patient preferences, and interdisciplinary collaboration. By balancing multiple conflicting scripts, the team facilitated a course of care aligned with the patient’s values, ultimately ensuring a patient-centered approach despite the inherent uncertainties. The use of iterative re-framing and the care team’s ability to remain flexible as new data emerged serves as a model for handling dynamic, evolving cases that require reevaluation of initial management goals.

## Clinical Teaching Points

1. The differential diagnosis for brown urine should include pigments from both exogenous (food, medications) and endogenous (myoglobin, blood breakdown products) sources. Careful review of urinalysis with microscopy can reveal helpful information such as proteinuria, casts, or dysmorphic cells that can suggest particular etiologies.^11^
2. ANCA vasculitis and RCC have potentially conflicting management pathways as the immunosuppression required to treat active vasculitis may impact the efficacy of the immunotherapy used to treat RCC. Immunosuppression theoretically risks progression of RCC, while cancer-directed immunotherapy might worsen ANCA vasculitis.^12^
3. In the presence of diagnoses with conflicting management scripts, incorporating interdisciplinary collaboration and patients’ values and preferences will help identify an optimal treatment approach.
